# Comparative Evaluation of Cost-Effectiveness, Clinical and Microbiological Parameters of Systemic Antibiotics Versus Local Drug Delivery in Aggressive Periodontitis

**DOI:** 10.7759/cureus.20985

**Published:** 2022-01-06

**Authors:** Brij Nandan, Debarchan Barman Roy, Vandana A Pant, Vandana Gupta, Upendra Bhaduria, Harpreet Kaur, Ojas Gupta

**Affiliations:** 1 Department of Periodontology, Sudha Rastogi College of Dental Sciences and Research, Faridabad, IND; 2 Medicine, All India Institute of Medical Sciences, New Delhi, IND; 3 Department of Periodontology, Babu Banarasi Das College of Dental Sciences, Lucknow, IND; 4 Division of Periodontics, Center for Dental Education and Research, All India Institute of Medical Sciences, New Delhi, IND; 5 Department of Community Dentistry, Centre for Dental Education and Research, All India Institute of Medical Sciences, New Delhi, IND; 6 Division of Oral Pathology and Microbiology, Centre for Dental Education and Research, All India Institute of Medical Sciences, New Delhi, IND; 7 Pathology, Institute of Medical Sciences, Banaras Hindu University, Varanasi, IND

**Keywords:** antibiotics, cost-effectiveness, porphyromonas gingivalis, aggregatibacter actinomycetemcomitans, aggressive periodontitis

## Abstract

Background: Periodontitis is a biofilm-associated inflammatory disease of the periodontium in which microbial component contributes to its initiation that mainly includes chronic periodontitis and aggressive periodontitis (AgP).

Aim: The present study aimed to ascertain a cost-effective treatment approach in AgP with minimal side effects by comparing systemic antibiotics (SA) and local drug delivery (LDD) as an adjunct to scaling and root planning (SRP). Furthermore, the objectives were to analyze its effect on clinical and microbial parameters.

Materials and Methods: The participants were randomly divided into two groups A and B with eleven participants each. Group A was administered with Chlosite Gel [LDD] whereas group B with amoxicillin (AMX) 500 mg + metronidazole (MTZ) 400 mg [SA] thrice daily for 14 days. Clinical parameters such as plaque index (PI), gingival index (GI), clinical attachment level (CAL), and pocket probing depth (PPD)] were recorded at baseline and three months postoperatively. Microbiological parameters i.e *Aggregatibacter actinomycetemcomitans* (Aa) and *Porphyromonas gingivalis* (Pg) counts were also measured at baseline and after three months.

Results: Statistically significant difference was observed in all clinical parameters i.e. PI, GI, CAL, and PPD after three months follow-up in groups A and B. Reduced bacterial load i.e. Aa and Pg was significant at three months in both the groups. However, on comparing the total expenditure of both the groups, group A ranged from 920-1480 ₹ while in group B it ranged from 330-360 ₹. Hence, lower expenditure in group B (3.7 fold) compared to group A was evident.

Conclusions: Cost-effective approach recommended in the present study is mainly to promote awareness among periodontal patients in the public and private sectors, who refuse to get dental treatment due to fear of high expenditure. This can be attained by incorporating SA as an alternative to LDD in AgP patients.

## Introduction

Periodontitis is a biofilm-associated inflammatory disease of the periodontium in which microbial component contributes to its initiation that mainly includes chronic periodontitis and aggressive periodontitis (AgP) [1]. Previously, AgP was considered a separate disease because of its aggressive nature, location of the lesion, familial tendencies, and thinness of subgingival biofilm [2]. However, according to the 2017 world workshop classification, AgP was grouped under a single category “periodontitis” based on staging and grading system [3]. AgP displays an inadequate host response to periodontopathogenic bacteria that is ascribed to increased expression of a wide variety of immunological and genetic risk factors [4]. Microbial flora in such patients is persistent and recalcitrant even after scaling and root planing (SRP). As a result, they penetrate deep into the connective tissue through the gingival sulcus which leads to another perspective of pathogenesis of AgP [5]. Periodontopathogens mainly responsible for AgP are *Aggregatibacter actinomycetemcomitans* (Aa) and *Porphyromonas gingivalis* (Pg). Hence incorporating systemic antibiotics (SA) as an adjunct to SRP in the treatment of AgP has been reported to be effective in reducing the microbial load from the tissue [6].

The association of metronidazole (MTZ) and amoxicillin (AMX) in the treatment of AgP has gained recognition worldwide. In addition to their therapeutic benefits, they have numerous adverse effects i.e bacterial resistance, allergic reactions, and abdominal discomfort. MTZ causes metallic taste, cramps, nausea, vomiting, headache, and gastrointestinal disturbances [7]. Thus to override the shortcomings, Goodson et al. were the first to propose the concept of local drug delivery (LDD); through this route, the drug reaches the base of the pocket and is maintained for an adequate period of time for the required therapeutic effect [8]. The advantage of LDD is that it is required in very low concentration along with being site-specific and overcomes the major disadvantage of being overpriced [9]. Therefore in developing countries like India, expensive treatments are neglected by most individuals especially when it is related to the oral cavity and hence understanding of cost-effectiveness becomes of utmost importance. The present study was thus primarily carried out to evaluate the cost-effective treatment for AgP with minimal side effects. Additionally, the study also analyzed the effects on clinical and microbial parameters by comparing SA and LDD as an adjunct to SRP.

## Materials and methods

In this study, 22 systemically healthy human volunteers coming to the routine outpatient department (OPD) were included to determine the cost-effectiveness between SA and LDD in patients of AgP. The study was approved by the institutional ethical committee of Babu Banarsi Das College of Dental Science, Lucknow, India, with reference number BBDCODS/17/2014. The participants were explained about the procedure and written consent was obtained prior to the start of the study. The inclusion criteria consisted of participants aged 25-55 years diagnosed with AgP with pocket probing depth (PPD) and clinical attachment level (CAL) of >4 mm and <6 mm. Volunteers with systemic disease, pregnant and lactating women, and those allergic to AMX/MTZ were excluded from the study. The participants were divided into two groups of 11 participants each. The first group was administered with Chlosite Gel (Ghimas SpA, Bologna, Italy) [LDD] and the second group with AMX (500 mg) + MTZ (400 mg) [SA]. Clinical parameters, plaque index (PI), gingival index (GI), CAL, and PPD, were recorded at baseline and three months postoperatively.

Microbiological analysis

The selected lesions and the adjacent teeth were isolated with cotton rolls. The supragingival plaque was removed with a sterile scaler and the subgingival plaque was removed carefully by a Gracey curette. The sample was then transported in an Eppendorf tube (Eppendorf India, Chennai, India) containing LURIA BERTANI (LB) solution (Sisco Research Laboratory, Mumbai, India) to the Central Drug Research Institute (CDRI), Lucknow. The participants subsequently underwent phase-I therapy including oral hygiene instructions. In group A, Chlosite Gel as the LDD system was administered in the deepest pocket quadrant and group B with AMX + MTZ [SA] thrice daily for 14 days.

Both the groups were re-evaluated after every month and SRP was done as and when required during the maintenance phase. After three months, PI, GI, PPD, and CAL were recorded and the subgingival plaque samples were again submitted for microbiological analysis for both the groups.

Microbiological samples were incubated for 24 hrs. The culture plate was prepared in CDRI by using a plaque sample. A selective medium for Aa was prepared by adding 200 µg/mL of vancomycin and 10 U/mL of bacitracin to *Aggregatibacter actinomycetemcomitans* growth medium (AAGM), which contained dextrose, sodium bicarbonate, trypticase soy, yeast extract, and agar [10]. The selective medium for Pg was prepared by making Colombia Agar which was supplemented with sheep blood, bacitracin, colistin, and nalidixic acid. The bacterial count was done by 10 fold dilution and plating method at baseline and three months postoperatively [11]. Polymerase Chain Reaction (PCR) was used as a confirmation test of Aa and results of the PCR amplification were analyzed by electrophoresis in a 1% agarose gel. The presence of Aa was determined as a distinct band of 547 base pairs.

Statistical analysis

The data collected were subjected to statistical analysis. The normality of the data was assessed using Shapiro-Wilk’s test and homogeneity of variance between groups by Levene’s test. Repeated measures two-factor analysis of variance (ANOVA) and Tukey’s post hoc test was used to determine the difference between the variables. A two-tailed p-value less than 0.05 (p<0.05) was considered statistically significant.

## Results

At baseline and three months, there was no significant difference in mean PI, GI, CAL, and PPD value between group A and group B; however, a statistically significant difference was seen between baseline and three months for all the parameters in group A and for gingival index and clinical attachment level in group B (Table 1).

**Table 1:** Comparison of mean plaque index, gingival index, clinical attachment level and mean probing depth Comparison in terms of (Mean ± SD) and (p-value) between group A and group B at baseline and 3 months

Periods	Group A	Group B	p-value
Mean plaque index			
Baseline	1.54 ± 0.15	1.55 ± 0.14	0.890
3 months	1.07 ± 0.15	1.11 ± 0.13	0.502
p-value	0.003	0.203	-
Mean gingival index			
Baseline	1.42 ± 0.18	1.43 ± 0.12	0.832
3 months	1.06 ± 0.16	1.12 ± 0.19	0.461
p-value	0.008	0.007	-
Mean clinical attachment level			
Baseline	3.43 ± 1.18	3.57 ± 1.21	0.778
3 months	2.41 ± 0.77	2.76 ± 0.95	0.348
p-value	0.001	0.001	-
Mean pocket probing depth			
Baseline	2.50 ± 0.25	2.55 ± 0.14	0.589
3 months	1.81 ± 0.18	1.88 ± 0.18	0.314
p-value	0.002	0.127	-

At baseline, the count of Aa was higher in group A when compared to group B. After three months of follow-up, the count of Aa decreased in both groups. However, a significant decrease was reported in group A when compared with group B. The mean bacterial count of Pg also decreased after three months of follow-up; however, no significant difference was reported in both the groups (Table 2).

**Table 2:** Mean bacterial count of Aggregatibacter actinomycetemcomitans and Porphyromonas gingivalis (Mean ± SD) and (p-value) are compared between group A and group B at baseline and 3 months

Periods	Group A	Group B	p-value
Mean bacterial count of Aggregatibacter actinomycetemcomitans			
Baseline	187545 ± 116148	287455 ± 583301	0.584
3 months	35000 ± 42584	30391 ± 45105	0.808
p-value	0.001	0.173	-
Mean bacterial count of Porphyromonas gingivalis			
Baseline	607273 ± 968794	509455 ± 743305	0.793
3 months	50727 ± 90782	24645 ± 37443	0.389
p-value	0.068	0.052	-

**Figure 1:**
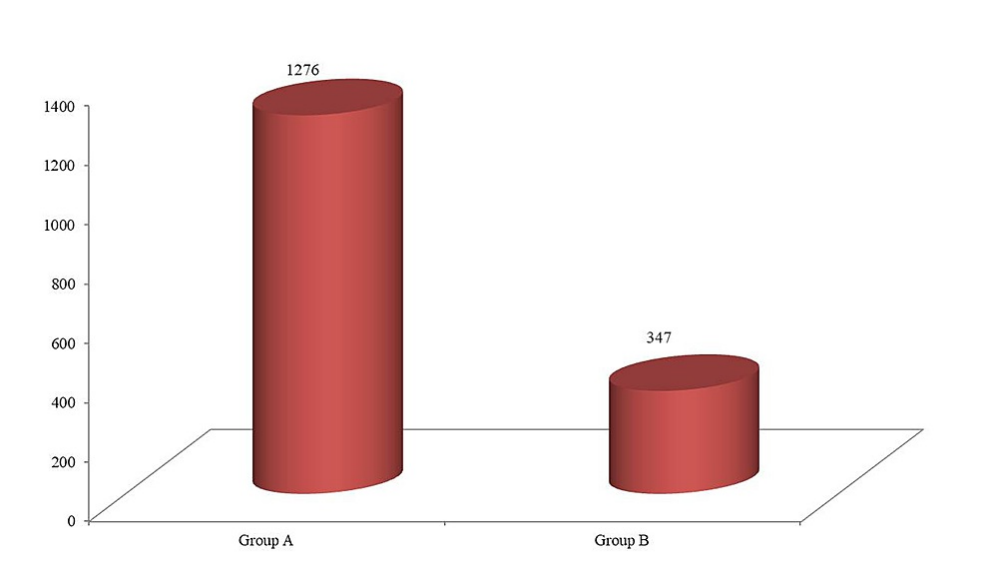
Comparison of total drug cost incurred between group A and group B

The total drug cost incurred in group A ranged from 920-1480 ₹ with mean cost being 1276 ± 181 ₹ while in group B the cost incurred ranged from 330-360 ₹ with mean cost being 347 ± 10 ₹. The mean total expenditure of group B was significantly lower than group A (Figure 1).

The mean total drug cost of group B was significantly lower than group A (p-value = .001) (Table 3). 

**Table 3:** Comparison of the total cost incurred between group A and group B Numbers in parenthesis indicate the range (min-max) t-value: Outcomes values of t-test

Group A	Group B	t-value	p-value
1276 ± 181 (920-1480)	347 ± 10 (330-360)	17.00	.001

## Discussion

Evidence reported the clinical and microbiological outcomes of SA and LDD in AgP [12-14]. However, to the best of our knowledge, the present study is the first that has evaluated the comparative effect of LDD and SA on the clinical and microbial profile as well as analyzing the cost distribution. The monitory benefit of this cost-effective analysis is an approach to avail oral health care in both public and private sectors from being unjustly neglected. Our data indicated that both the therapies were comparable in reducing the periodontal pathogenic species along with gain in clinical parameters over time.

Present findings with the improvement of clinical parameters in group B were consistent with the result of Guerrero et al. where PI, GI, PPD, and CAL decreased at two and six months from baseline [12]. However, in group A, the decrease in clinical parameters was in accordance with the clinical trial conducted by Sakellari et al. who evaluated the effects of LDD in the form of tetracycline fibers and suggested its adjunctive use to improve clinical response at two and six months after treatment [15]. A similar observation by Kaner et al. in 2007 compared LDD chlorhexidine chip (CHC) and SA as an adjunct to SRP in AgP and concluded SA to be more efficacious in clinical outcomes than CHC in an entire observation period of three and six months [13]. In contrast, Badersten et al. concluded that PPD was increased when LDD was administered in suppurating sites that resulted in attachment loss [16].

The effect of SRP alone on subgingival microflora has been reported in the literature that reduces microbial load and results in a shift towards a more health compatible microflora [17]. However, there are conflicting reports regarding the ability of SRP to eradicate or suppress periodontal pathogens mainly Aa and Pg. Hence, particulars from the present study agree that viable microbial counts of Aa and Pg in group A decreased significantly from baseline to three months in AgP. Nevertheless to our knowledge, similar observation has not been found in the literature in AgP patients, although LDD in form of CHC in adult periodontitis has proven no additional antimicrobial benefit when compared to SRP alone [18].

Similarly, in group B this therapy led to a significant reduction in viable counts of Aa and Pg which is in concordance with the findings of Mestnik et al. [19]. Also, evidence suggests a possible synergistic effect of (AMX + MTZ) drugs in inhibiting Aa [20]. In conjunction with the beneficial clinical and microbiological results observed in the present study, it was also interesting to observe that none of the subjects from groups A and B have presented any adverse effects.

Lastly in our data analysis, the total expenditure incurred in group A ranged from 920-1480 ₹while in group B it ranged from 330-360₹. The mean total expenditure observed for group B was quite lower than group A (3.7 fold or 72.8%) and the difference was statistically significant. In accordance with our study, De Lissovoy et al. in a 12-month, prospective clinical trial specifically examined and captured, for the first time, the full economic costs of periodontal treatment [21].

Limitations

Limitations of the current study are the short-term evaluation period and recruitment of a small percentage of AgP patients. Indeed longitudinal assessment of these patients is important in order to determine whether and to what extent LDD and SA produce a beneficial change in clinical and microbial profile over time.

## Conclusions

The cost-effective approach recommended in the present study is mainly to promote awareness among periodontal patients who refuse to get dental treatment due to fear of high expenditure. Further, this can be emphasized by incorporating antimicrobials as an alternative to LDD in AgP cases. This will definitely aid the clinician to achieve a better outcome in the maintenance phase itself, even in those patients who cannot afford the high expense of various treatment approaches. However, further studies with a larger sample of aggressive periodontitis are mandatory to show the clear equivalence of both treatment modalities.
